# Serious Complications of Intraosseous Access during Infant Resuscitation

**DOI:** 10.1055/s-0038-1661407

**Published:** 2018-07-19

**Authors:** Jiri Molacek, Karel Houdek, Václav Opatrný, Jiri Fremuth, Lumir Sasek, Inka Treskova, Vladislav Treska

**Affiliations:** 1 Department of Vascular Surgery, University Hospital in Pilsen, Pilsen, Czech Republic; 2 Department of Pediatric Intensive Care Unit, University Hospital in Pilsen, Pilsen, Czech Republic; 3 Department of Plastic Surgery, University Hospital in Pilsen, Pilsen, Czech Republic

**Keywords:** intraosseous access, limb amputation, compartment syndrome

## Abstract

We report on a 2.5-month-old infant with ischemia of the left leg and compartment following intraosseous needle application during resuscitation. Unfortunately, this event led to major limb amputation. The cause, mechanism, and prevention of this severe complication are discussed in this article.

## Introduction

Establishing vascular access during cardiopulmonary resuscitation is essential due to the need for immediate administration of drugs and the possibility of volume therapy. Routine peripheral vein cannulation may be difficult in several situations, including extensive multiple trauma with hemorrhagic shock and centralized circulation or extensive burns. One of the possible alternatives is intraosseous (IO) access, a relatively quick and easy option for the administration of drugs or larger amounts of fluid. The authors present a very serious complication in this IO access that occurred during resuscitation. The aim of the work presented here is to point out that even a commonly used method for establishing vascular access can lead to very severe complications.

## Case Report

**Fig. 1:**
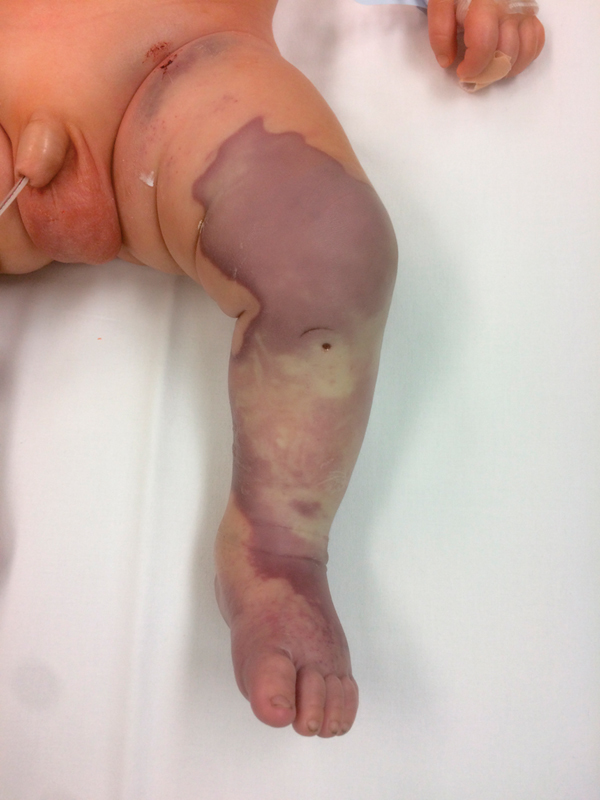
Left lower limb on admission, a visible insertion site after intraosseous access.

**Fig. 2:**
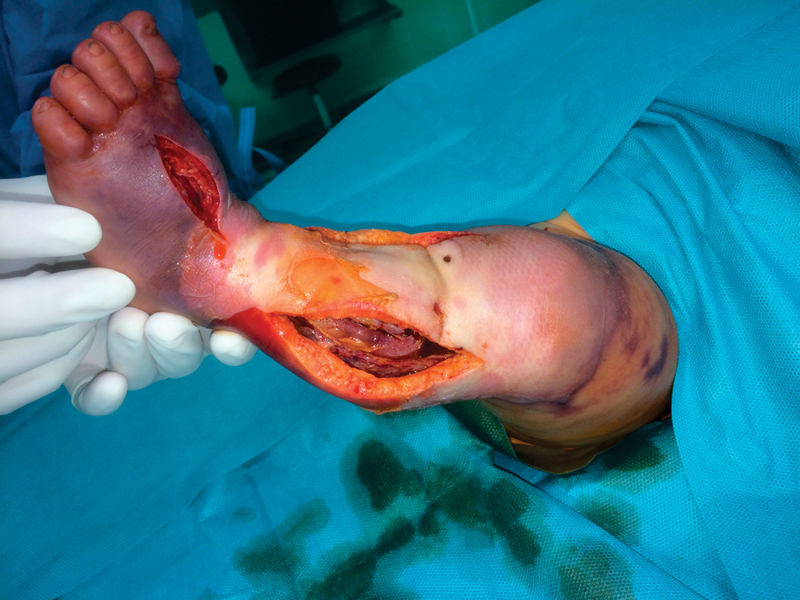
Fasciectomies performed.

**Fig. 3:**
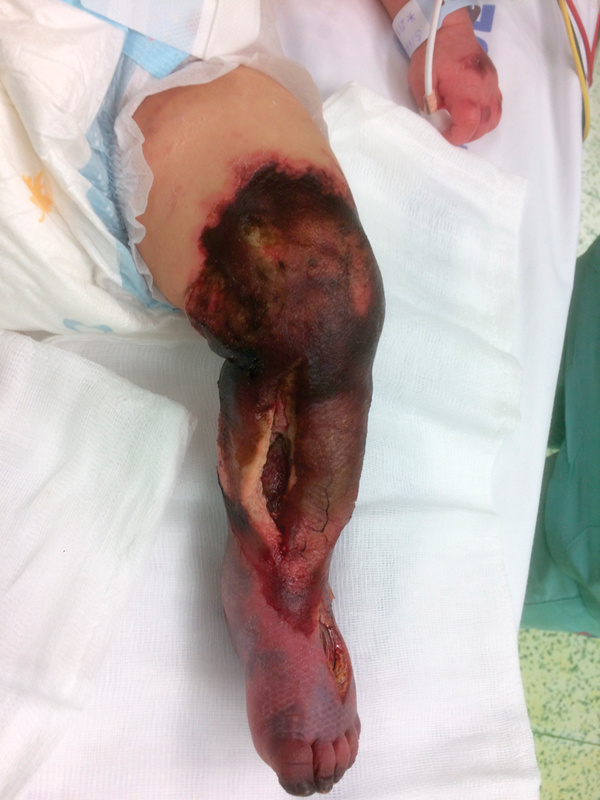
Limb gangrene.

**Fig. 4:**
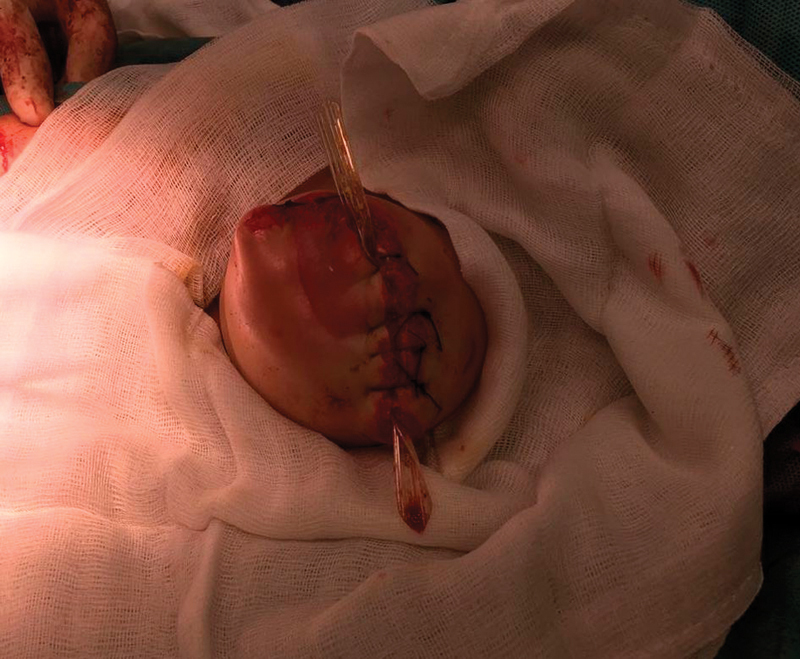
Above knee amputation.

The emergency medical service (EMS) was called by the parents of a 2.5-month-old infant. The parents of a previously healthy infant reported a sudden loss of consciousness and respiratory arrest. The ambulance crew arrived 8 minutes after the initial call and found the infant in cardiac arrest. The parents had started resuscitation according to instructions from the EMS dispatcher over the phone (∼9 minutes). Cardiopulmonary resuscitation was immediately commenced by the EMS physician. After an unsuccessful attempt to insert a peripheral venous catheter, IO access into the left tibial tuberosity was performed to provide vascular access. The heart rate was restored ∼2 minutes after the start of medical resuscitation. The infant was intubated using a nasotracheal tube and brought to the emergency unit and later to the intensive care and resuscitation unit (ICRU) of the Pediatric Department at the University Hospital. Upon admission to the ICRU, the patient was described as being nonreactive, with mydriatic pupils and unmeasurable blood pressure. Inotropic support (initiated at the scene) was continued with a high-dose adrenaline. During admission, a swelling of the left lower limb (the side with inserted IO cannula) was immediately appreciated. Due to the suspected extravasation and extramedullary distribution of catecholamines and crystalloids, identical IO access was established on the lower right limb and the IO needle on the left leg was removed. A central venous catheter was later introduced via the left subclavian vein. Baseline laboratory tests revealed leukocytosis with a left shift, procalcitonin elevation, laboratory signs of severe hepatopathy and coagulopathy. Clinical signs of multiple organ failure were present. After ∼120 minutes, the infant's condition was stabilized, and acute computed tomography scan (CT brain scan + the whole body CT scan) was performed, which revealed extensive thrombosis of the cerebral veins and generalized visceral and limb vasoconstriction. About 3 hours after admission, a vascular surgeon was contacted due to increased edema and livid discoloration of the lower left leg. The vascular surgeon evaluated the limb as a combination of iatrogenic compartment syndrome (due to extravasation of catecholamines and crystalloids) and limb ischemia. He described a hard swelling of the entire lower leg and livid discoloration of almost the entire limb ( Fig. 1 ). Urgent fasciotomy of all muscle beds was indicated as the only way to save the limb with two incisions as usual from two skin incisions (medially and laterally to the tibial edge [ Fig. 2] ). The muscles extruded immediately after cutting the fascia. Large areas of inferior and posterior groups of the muscles were grayish and not likely vital. An incision was also made in the dorsal foot area, where marked swelling was also present. The fasciotomy wounds were covered with a lubricated, moist dressing. A dressing change was scheduled at 48 hours post fasciotomy. During these 2 days, the infant remained in very serious condition, and was on permanent circulatory support. An EEG scan revealed a severely abnormal electroencephalogram, with a very depressed background with periodic occurrence of burst suppression EEG pattern. The EEG pattern corresponds to an unfavorable prognosis. Two days later, the dressing on the lower left limb was changed and clearly necrotic tissues were removed. The vitality of most muscles was still very unlikely. In the following period, the patient's condition stabilized, but the cause of circulatory arrest remained unknown and severe consciousness disorder continued. The dressing on the lower left leg was changed every 2 days in the operating room. Full conservative anticoagulation and vasodilation therapy were still indicated. The finding on the limb gradually deteriorated, and the patient developed skin necrosis ( Fig. 3 ) that was gradually removed along with a part of the muscles. While in comprehensive intensive care, the infant's overall condition and consciousness disorder improved, but the local finding on the left lower limb significantly progressed and resulted in above knee amputation on day 19 after the admission ( Fig. 4 ). The amputation stump healed without complications, the overall condition continued to improve, and the infant was discharged to home care on day 51 after the admission. During the hospitalization, no clear explanation was found for the primary cause of the cardiac arrest. No congenital developmental defects and no mitochondrial defects were found. As a result of postischemic brain damage, recurrent epileptic attacks occur. A magnetic resonance imaging scan of the brain performed (1 month after the event) showed clear postischemic changes. The extent of brain tissue damage will be revealed during further development of the infant.

## Discussion

Immediate intravenous (IV) access for the administration of drugs or infusions is absolutely necessary during resuscitation and more generally in urgent or intensive medicine. Access to the bone marrow may be beneficial when IV access cannot be established for any reason. The IO administration route was first introduced by Drinker in 1922. Through IO access, infusions may be administered at the same rate as IV administration. Some intensive care guidelines recommend using IO access after repeated failure of attempts to establish IV access. During the resuscitation, IO access can be established even after the first failed IV access attempt. 1 Several systems available on the market differ by method (punching, drilling, etc.). The most common sites for intramedullary access are the proximal tibia (suitable for children), humeral head (more common in adults), or sternal manubrium. The most commonly reported complications of IO access are infection at the injection site, which may result in severe osteomyelitis, damage to the growth plate, and fat embolism, 2 which have been reported in adults. Dislocation of the needle followed by extravasation should also be considered (analogy of IV cannula dislocation 3 ). In this case report, very severe complications occurred after the malposition of the IO needle during the resuscitation, and subsequent extravasation of both the crystalloid infusion and inotropic support. In our opinion, two mechanisms contributed to the development of these serious findings in our case: a combination of a mechanical insult due to extravasation of the crystalloid and the vasoconstrictor mechanism. In the infant patient, total visceral and limb vasoconstriction occurred. The local influence of adrenalin leakage further complicated the situation. In the medical facility, the malposition of the IO needle was immediately detected and the needle was removed. Complications associated with the dislocation of the IO needle are more likely in obese people. Another risk factor is the necessity of extensive manipulation of the patient, such as during the resuscitation. The precise identification of the site of insertion is essential to avoid any complications of IO access. The most commonly used access site is the proximal tibial region; this is located 1 to 2 cm medially to the tibial tuberosity in adults and 1 cm medially and 1 cm distally to the tibial tuberosity in children. For a proximal humerus, this is located ∼2 cm above the surgical neck. The selection of an appropriate intramedullary needle size is also crucial. In newborns and infants/toddlers, limb compartment syndrome is very rare and is always associated with trauma or some invasive intervention. The most common site of occurrence is on the leg. 4 5 6 7 Late diagnosis of this complication is not uncommon and logically has worse outcomes. The consequences of this complication may be very serious; the loss of the limb is rare. The infant patient in our case, unfortunately, developed a combination of limb ischemia due to adrenaline extravasation, centralization due to circulatory instability, and compartment syndrome due to extravasation of crystalloids. The question is how to prevent such serious consequences in this complication of IO access, where early diagnosis and immediate intervention are more difficult due to the overall poor condition of the patient. In our case, even very fast diagnosis and surgical intervention could not prevent irreversible damage to the soft tissues of the limb.

## Notes

New Insights and the Importance for the Pediatric Surgeon Common use of intraosseous needle in newborn can have severe complication. Needle malposition can cause severe compartment syndrome and limb ischemia.
